# Single-cell RNA sequencing reveals distinct transcriptional features of the purinergic signaling in mouse trigeminal ganglion

**DOI:** 10.3389/fnmol.2022.1038539

**Published:** 2022-10-13

**Authors:** Shilin Jia, JinYue Liu, Yanhao Chu, Qing Liu, Lijia Mai, Wenguo Fan

**Affiliations:** ^1^Hospital of Stomatology, Guanghua School of Stomatology, Sun Yat-sen University, Guangzhou, China; ^2^Guangdong Provincial Key Laboratory of Stomatology, Guangzhou, China; ^3^Paediatric Dentistry and Orthodontics, Faculty of Dentistry, The University of Hong Kong, Hong Kong, Hong Kong SAR, China

**Keywords:** purinergic signaling, purinergic receptor, adenosine triphosphate, trigeminal ganglion, trigeminal neurons, single-cell RNA sequencing

## Abstract

Trigeminal ganglion (TG) is the first station of sensory pathways in the orofacial region. The TG neurons communicate with satellite glial cells (SGCs), macrophages and other cells forming a functional unit that is responsible for processing of orofacial sensory information. Purinergic signaling, one of the most widespread autocrine and paracrine pathways, plays a crucial role in intercellular communication. The multidirectional action of purinergic signaling in different cell types contributes to the neuromodulation and orofacial sensation. To fully understand the purinergic signaling in these processes, it is essential to determine the shared and unique expression patterns of genes associated with purinergic signaling in different cell types. Here, we performed single-cell RNA sequencing of 22,969 cells isolated from normal mouse TGs. We identified 18 distinct cell populations, including 6 neuron subpopulations, 3 glial subpopulations, 7 immune cell subpopulations, fibroblasts, and endothelial cells. We also revealed the transcriptional features of genes associated with purinergic signaling, including purinergic receptors, extracellular adenosine triphosphate (eATP) release channels, eATP metabolism-associated enzymes, and eATP transporters in each cell type. Our results have important implications for understanding and predicting the cell type-specific roles of the purinergic signaling in orofacial signal processing in the trigeminal primary sensory system.

## Introduction

The somatosensory system processes mechanical, thermal, and chemical information about the internal physiological state and the environment to provide organisms “feel” touch, temperature, pain, itch, and more (Dubin and Patapoutian, 2010; Wu et al., 2017; Dong and Dong, 2018; Yin and Lee, 2020). The sensory neurons of the trigeminal ganglion (TG, a cranial analog of the dorsal root ganglia, DRG) are the primary somatosensory receptors, innervating the orofacial region, and conveying signals to the central nervous system (Messlinger and Russo, 2019; Delmas and Coste, 2020). A variety of neurotransmitters and mediators associated with nociception are present in neurons and non-neuronal cells, such as satellite glial cells (SGCs), Schwann cells (SCs) and macrophages in the peripheral nervous system (PNS), through which crosstalk between these cells plays a vital role in the etiology and pathogenesis of pain (Goto et al., 2017; Wang et al., 2018; Messlinger et al., 2020; Iwai et al., 2021).

Purinergic signaling is one of the most critical autocrine/paracrine signaling pathways involved in intercellular communication. It is a versatile system that includes extracellular adenosine triphosphate (eATP), purinergic receptors, eATP transport pathways, and eATP metabolism-associated enzymes (Antonioli et al., 2019). It is now recognized that the purinergic signaling acts as a modulatory system in the peripheral somatosensory system and is involved in the regulation of orofacial pain (Shinoda et al., 2007; Murasaki et al., 2013; Lin et al., 2019). However, the expression of the components associated with purinergic signaling in each cell type of TG has not yet been characterized in detail.

Single-cell RNA sequencing (scRNA-seq) has emerged as a powerful tool for cell-type heterogeneity identification and cell type-specific gene expression analysis (Kolodziejczyk et al., 2015). Here, we aimed to identify distinct cellular populations in TG and describe the transcriptional features of the genes associated with the purinergic signaling in each cell type. We provided a comprehensive transcriptomic atlas of the purinergic signaling in TG. Our findings contribute to the understanding of the purinergic signaling mechanism of neuromodulation and neurotransmission.

## Materials and methods

### Animals

Male C57BL/6 mice weighing 20–25 g (5–8 weeks) were housed in cages under standard conditions (12-h light/dark cycle) with access to food and water *ad libitum*. All experimental procedures were performed in accordance with relevant guidelines established by the Animal Care Committee for the Care and Use of Laboratory Animals of Sun Yat-sen University (Guangzhou, Guangdong Province, China; Ethical clearance No. SYSU-IACUC-2020-000245).

### Preparation of single-cell suspension from trigeminal ganglion

Five mice were euthanized by 4% isoflurane inhalation and decapitation. Bilateral TGs were carefully harvested (*n* = 10) and then were quickly chopped into small pieces and dissociated using a papain dissociation system (Worthington, Lakewood, NJ, USA, 20 units/ml papain, 0.005% DNase) according to the manufacturer’s instructions. The mixture was incubated at a 37°C water bath for 1 h with constant agitation, and then the cloudy suspension was collected and centrifuged at 300 g for 5 min at room temperature. The supernatant was discarded, and the cell pellets were resuspended in a DNase/papain-inhibitor solution (2.7 ml EBSS, 300 μl papain-inhibitor and 150 μl DNase). The obtained cell suspension was purified by discontinuous density gradient centrifugation (70 g for 6 min), and then the cell pellets were resuspended in DMEM to obtain a single-cell suspension. All solutions were equilibrated with 95% O_2_ and 5% CO_2_ throughout the procedure.

### RNA library construction and sequencing

scRNA-seq libraries were constructed using the BD Rhapsody™ Single-Cell Analysis System according to the manufacturer’s instructions. Briefly, single cells were captured and lysed with barcoded beads on an array of microwells. The mRNA was enriched by the oligo(dT) beads in the microwell and each mRNA molecule was tagged with a unique molecular identifier (UMI) and cell label (Birey et al., 2017). After reverse transcription of the tagged mRNA and amplification, the cDNA libraries were constructed. Libraries were sequenced on the Illumina NovaSeq platform. The sequencing data (FASTQ file) were processed by the BD Rhapsody analysis pipeline on Seven Bridges^1^ to obtain the gene expression matrix (ST file). Sequence data were submitted to the Gene Expression Omnibus (GEO) database (GSE213105).

### Preprocessing of gene expression matrix

Data preprocessing was performed using Seurat R package v4.0.2. As quality controls, cells with more than 15% mitochondrial gene proportion, less than 300 detected genes, or less than 500 UMIs were discarded. The filtered count matrix was normalized with a scale factor of 10,000 and subsequently natural-log transformed using Seurat’s NormalizeData function. The normalized count matrix was further scaled with Seurat’s ScaleData function.

### Data integration

To ensure our results are reliable, the same analysis pipeline was performed in another TG sample (*n* = 6) derived from the GEO database (GSE186421).^2^ Integration of the two datasets was performed using canonical correlation analysis (CCA) algorithm (Stuart et al., 2019). The integrated matrix was processed as described above for subsequent analysis.

### Dimensionality reduction and clustering analysis

The top 3,000 highly variable genes were identified using Seurat’s FindVariableFeatures function, and Principal component (PC) analysis was subsequently performed using Seurat’s RunPCA function. Then, the standard deviations of the PCs were visualized by the ElbowPlot function, and the top 20 PCs were selected according to elbow position for non-linear dimensionality reduction and visualization (Uniform Manifold Approximation and Projection, UMAP). Unsupervised identification of clusters was performed using the FindClusters function (an algorithm based on Shared Nearest Neighbor construction and modularity optimization).

### Cell type annotation

Cell types were manually annotated based on marker genes of each cluster, and the manual annotations were verified by automated cell annotation using SingleR R package v1.4.1. Cluster-specific marker genes were identified using the FindAllMarkers function with parameters: logfc.threshold = 1.5 and min.pct = 0.6. Gene symbols and corresponding protein names can be found in Supplementary Table 1. The reference databases used for manual annotation were obtained from CellMarker^3^ and PanglaoDB^4^ (Franzén et al., 2019; Zhang et al., 2019). The MouseRNAseqData database was utilized for automated annotation using celldex R package v1.0.0.

### Differential gene expression analysis

Differential gene expression analysis among distinct cell populations was performed using the FindMarkers function with parameters: logfc.threshold = 0.2 and min.pct = 0.05. Genes with a *p*-value less than 0.001 and log_2_ fold change(log_2_FC)more than 0.5 were considered significantly differentially expressed. log_2_FC was calculated through the following formula: log2FC = log2(*Average expression value.1* + 1)-log2(*Average expression value.2* + 1). *Average expression value.1* refers to the average expression of a gene in the indicated cell groups, and *Average expression value.2* refers to the average expression of this gene in another or more cell groups, which is usually set as all cells except the indicated cell population.

### Statistical analysis

Statistical analyses were performed using the FindMarkers or FindAllMarkers function of the Seurat R package. The significance of differential gene expression was assessed using the Wilcoxon Rank Sum test. Statistical significance was defined as a *P*-value less than 0.01.

## Results

### Single-cell transcriptomics reveal cell-type heterogeneity in trigeminal ganglion

**FIGURE 1:**
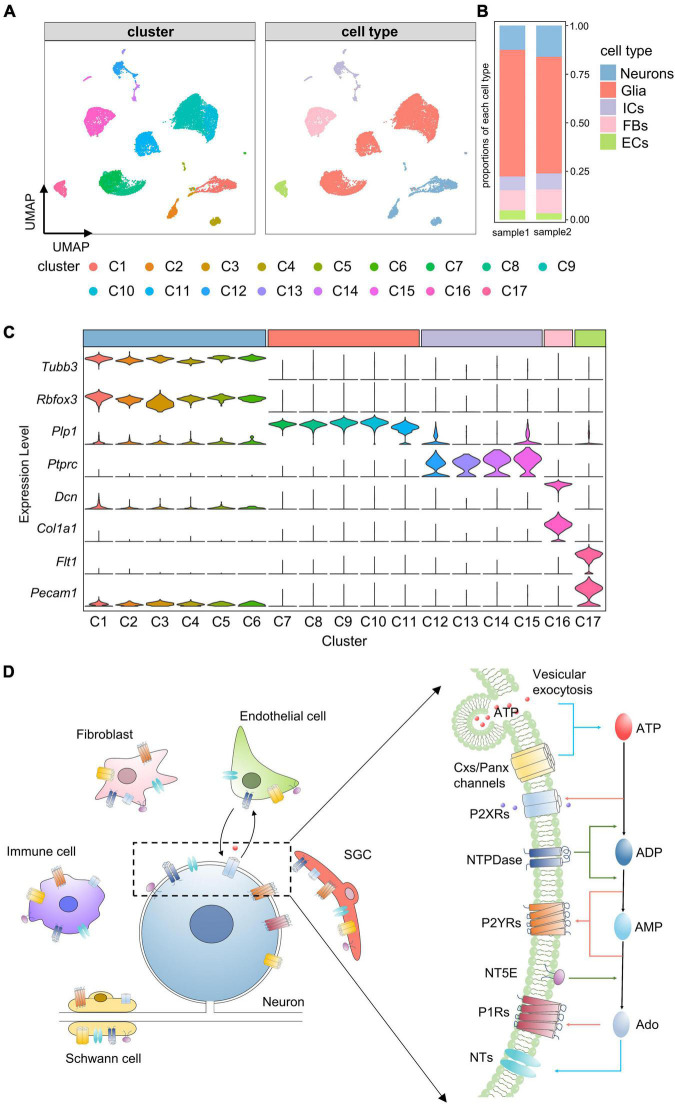
Identification of cell clusters and cell types in TG. **(A)** UMAP projection of cell clusters and cell types. Unsupervised cell clustering and cell type annotation identify 17 distinct cell clusters (C1–C17) and 5 cell types, including neurons, glial cells, immune cells, fibroblasts and endothelial cells. Each dot represents one cell; Each color represents one cluster or one cell type. **(B)** Bar plot showing proportions of each cell type in each TG sample. **(C)** Stacked violin plot showing the distribution of known marker genes (e.g., neuronal marker genes: *Tubb3* and *Rbfox3*) in each cluster. The width of the violin plots represents density distributions, the height represents gene expression levels [normalized (counts)], and each color represents one cluster. C1–C6 were identified as neurons by high expression of *Tubb3* and *Rbfox3*, C7–C11 were identified as glial cells by *Plp1*, C12-C15 were identified as immune cells by *Ptprc*, C16 was identified as fibroblasts by *Dcn* and *Col1a1*, C17 was identified as endothelial cells by *Flt1* and *Pecam1*. **(D)** Schematic illustration of purinergic signaling in TG. Extracellular ATP is released from TG cells *via* vesicular exocytosis and Cxs/Panx channels; ATP-activated P2XRs mediate non-selective cation influx; ATP-metabolism associated ecto-enzymes, such as NTPDase and NT5E, hydrolyze ATP to ADP, AMP and Ado; Both ADP and AMP activate P2YRs, and Ado activates P1Rs; In addition, NTs mediate the bidirectional diffusion of Ado between the cytoplasm and extracellular environment. UMAP, Uniform Manifold Approximation and Projection; Neu, neurons; Glia, glial cells; ICs, immune cells; FBs, fibroblasts; ECs, endothelial cells; ATP, adenosine triphosphate; P2XRs, P2X receptors; NTPDase, ectonucleoside triphosphate diphosphohydrolase; NT5E, Ecto-5’-nucleotidase; ADP, adenosine diphosphate; AMP, adenosine monophosphate; Ado, adenosine; P2YRs, P2Y receptors; P1Rs, P1 receptors; NTs, nucleoside transporters. SGC, Satellite glial cells.

To assess the cellular heterogeneity in TG, we prepared single cell suspensions of mouse TG and performed scRNA-seq. A total of 22,969 cells from two independent samples were sequenced. Following quality control (see section “Materials and methods”), a total of 16,294 cells were obtained and used for subsequent analyses. To identify cell clusters, we performed non-linear dimensionality reduction and unsupervised cell clustering using the Seurat R package. 17 distinct clusters (C1–17) were identified and visualized on a UMAP plot (Figure 1A). Then, these clusters were classified into 5 major cell types based on the expression of known marker genes, including neurons (*Tubb3* and *Rbfox3*), glial cells (*Plp1*), immune cells (*Ptprc*), fibroblasts (*Dcn* and *Col1a1*), and endothelial cells (*Flt1* and *Pecam1*) (Figures 1A,C). Among these cell types, the proportion of glial cells was the highest, followed by neurons and fibroblasts, and the proportions of each cell type were similar across the two samples (Figure 1B).

To describe the transcriptional features of the purinergic signaling in each cell type, we categorized genes associated with the purinergic signaling into four parts: genes encoding purinergic receptors, eATP release channels, eATP metabolism-associated enzymes, and ATP/adenosine transporters (Figure 1D). Gene symbols and corresponding protein names are shown in Supplementary Table 2. Expression rates [percentage of cells with positive expression (pos_pct)] and average expression levels (avg_exp) of these genes in different cell types were analyzed.

### Purinergic receptors

**FIGURE 2:**
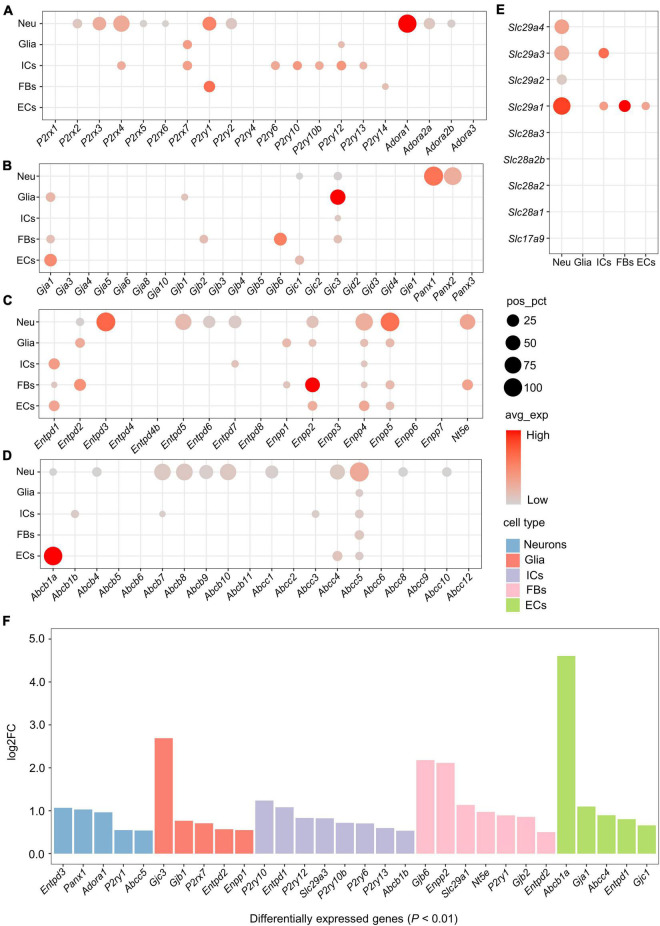
Expression of genes associated with purinergic signaling in distinct cell types. **(A–E)** Bubble plot showing expression of genes encoding purinergic receptors, ATP release channels, ATP metabolism-associated enzymes or ATP/adenosine transporters in the 5 cell types. Bubble size represents the percentage of cells with positive expression (pos_pct), and bubble color represents average normalized expression levels (avg_exp). (Bubbles with pos_pct < 5% are not shown). **(F)** Bar plot showing significant differences in the expression of genes associated with purinergic signaling among 5 cell types (Wilcoxon Rank Sum test; *P* < 0.01 was considered statistically significant). The bars are color-coded by cell types, and the bar height represents log2 fold change (log2FC). Genes with log2FC > 0.5 and *P* < 0.01 are considered to have significantly higher expression.

Among genes encoding ATP-gated P2X cation channels, five *P2rx* genes were expressed in more than 5% of neurons, including *P2rx2*-*P2rx6* (Figure 2A). Among these, *P2rx4* was the most widely expressed in the neurons (pos_pct = 64.2%), followed by *P2rx3* (pos_pct = 38.4%). Compared with the neurons, non-neuronal cells expressed relatively few *P2rx* family members. Glial cells positively expressed only *P2rx7* (pos_pct = 12.4%), while immune cells positively expressed *P2rx4* (pos_pct = 10.5%) and *P2rx7* (pos_pct = 13.3%). However, less than 5% of fibroblasts and endothelial cells expressed *P2rx* genes (Figure 2A). Moreover, differential gene expression analysis among the 5 cell types revealed that *P2rx7* was expressed significantly higher in glial cells (log_2_FC > 0.5 and *P* < 0.01; Figure 2F). Among genes encoding P2Y receptors, *P2ry1* and *P2ry2* were positively expressed in the neurons, *P2ry12* in glial cells, *P2ry6*, *P2ry10*, *P2ry10b*, *P2ry12*, and *P2ry13* in immune cells, and *P2ry1* and *P2ry14* in fibroblasts (pos_pct > 5%; Figure 2A). Moreover, *P2ry1* was expressed significantly greater in neurons and fibroblasts. Five *P2ry* genes including *P2ry6*, *P2ry10*, *P2ry10b*, *P2ry12*, and *P2ry13* were expressed more highly in immune cells than the other cells (log_2_FC > 0.5 and *P* < 0.01; Figures 2A,F).

The neuronal population showed positive expression of genes encoding adenosine receptors (P1 receptors), including *Adora1*, *Adora2a* and *Adora2b* (Figure 2A). Among these, *Adora1* was the most widely expressed (pos_pct = 86.8%), followed by *Adora2a* (pos_pct = 22.9%) and *Adora2b* (pos_pct = 8.9%). Expression of *Adora3* was only detected in a small number of immune cells (pos_pct = 1.87%) and neurons (pos_pct = 0.21%).

### Cxs and Panx channels

Both connexin hemichannels (Cxs) and Panx channels (Panxs) are transmembrane proteins, mediating the release of small signaling molecules such as ATP *via* their central pores (Taruno, 2018). Among genes encoding Cxs, *Gjc3* was positively expressed in all the cell types except endothelial cells, *Gja1* was positively expressed in glial cells, fibroblasts and endothelial cells, and *Gjc1* was positively expressed in neurons and endothelial cells (pos_pct > 5%; Figure 2B). In addition, glial cells expressed *Gjb1* positively, and fibroblasts expressed *Gjb2* and *Gjb6* positively. Differential gene expression analysis showed that *Gjb1* and *Gjc3* had significantly higher expression in glial cells, *Gjb2* and *Gjb6* in fibroblasts, and *Gja1* and *Gjc1* in endothelial cells (log_2_FC > 0.5 and *P* < 0.01; Figure 2F).

Panxs-encoding genes were exclusively expressed in neurons. *Panx1* was expressed in almost all neurons (pos_pct = 97.8%), *Panx2* was expressed in more than 80% of the neurons, whereas *Panx3* expressed less than 5% (Figure 2B). No positive expression of *Panx* family members was found in non-neuronal cells.

### Extracellular adenosine triphosphate metabolism-associated enzymes

eATP metabolism-associated enzymes include ectonucleoside triphosphate diphosphohydrolase (E-NTPDase, encoded by *Entpd*), ectonucleotide pyrophosphatase/phosphodiesterase (ENPP, encoded by *Enpp*) and Ecto-5’-nucleotidase (NT5E, encoded by *Nt5e*) (Yegutkin, 2014).

Five *Entpd* genes including *Entpd2*, *3*, *5*, *6*, and *Entpd7* were positively expressed in neurons (pos_pct > 5%), of which *Entpd3* and *Entpd5* were expressed in more than 50% of the neurons (pos_pct = 94.9% and 63.3%, respectively; Figure 2C). *Entpd1* was mainly expressed in non-neuronal cells, including immune cells, fibroblasts and endothelial cells. In addition, glial cells and fibroblasts expressed *Entpd2* positively, and immune cells expressed *Entpd7* positively. Moreover, differential gene expression analysis revealed that *Entpd3* was expressed significantly higher in neurons, *Entpd2* in glial cells and fibroblasts, *Entpd1* in immune cells and endothelial cells (log_2_FC > 0.5 and *P* < 0.01; Figure 2F).

Among genes encoding ENPP, *Enpp4* was positively expressed in all cell types (pos_pct > 5%), *Enpp2* and *Enpp5* were expressed in all cell types except immune cells, and *Enpp1* was expressed in glial cells and fibroblasts (Figure 2C). Both *Enpp4* and *Enpp5* were widely expressed in the neurons (pos_pct = 76.4% and 92.8%, respectively). Differential gene expression analysis showed that *Enpp1* had significantly higher expression in glial cells, and *Enpp2* in fibroblasts (log_2_FC > 0.5 and *P* < 0.01; Figure 2F). We also found that *Nt5e* was positively expressed in 56.0% of neurons and 20.0% of fibroblasts, whereas no positive expression of *Nt5e* was detected in glial cells, immune cells or endothelial cells.

### Adenosine triphosphate and adenosine transporters

ATP Binding Cassette transporters (ABCTs), including multidrug resistance protein (MDR, encoded by *Abcb*) and cystic fibrosis transmembrane conductance regulator (CFTR, encoded by *Abcc*), contribute to eATP release (Abraham et al., 1993; Reigada and Mitchell, 2005). Eleven ABCT-encoding genes were found to be positively expressed in neurons (pos_pct > 5%), of which *Abcb7*, *Abcb8*, *Abcb10*, *Abcc4*, and *Abcc5* were expressed in more than 50% of the neurons (Figure 2D). Among non-neuronal cells, all cell types expressed *Abcc5* positively (pos_pct > 5%). Immune cells expressed *Abcb1b*, *Abcb7*, and *Abcc3* positively, and endothelial cell expressed *Abcb1a* and *Abcc4* positively. Differential gene expression analysis revealed that *Abcc5* was significantly higher expressed in neurons, *Abcb1b* in immune cells, and *Abcb1a* in endothelial cells (log_2_FC > 0.5 and *P* < 0.01; Figure 2F).

Nucleoside transporters (NTs), including equilibrative NTs (ENTs, encoded by *Slc29a*) and concentrative NTs (CNTs, encoded by *Slc28a*), mediate the bidirectional diffusion of adenosine between the cytoplasm and extracellular environment (Kong et al., 2004). All *Slc29a* genes were positively expressed in neurons (pos_pct > 5%), of which *Slc29a1* was the most widely expressed (pos_pct = 76.9%), followed by *Slc29a3* (51.7%; Figure 2E). Among non-neuronal cells, immune cells expressed *Slc29a1* and *Slc29a3* positively, fibroblasts and endothelial cells expressed *Slc29a1* only, whereas no positively expression of these genes was detected in glial cells. Moreover, differential gene expression analysis showed that *Slc29a1* was significantly higher expressed in fibroblasts, and *Slc29a3* in immune cells (log_2_FC > 0.5 and *P* < 0.01; Figure 2F).

Vesicular nucleotide transporter (VNUT, encoded by *Slc17a9*) is responsible for the vesicular storage of ATP by membrane potential-dependent active transport (Sawada et al., 2008). However, no positive expression of *Slc17a9* (encoding VNUT) or *Slc28a* (encoding CNTs) was found in any of these cell types (pos_pct < 5%; Figure 2E).

### Expression of genes associated with purinergic signaling in neuronal subpopulations

**FIGURE 3:**
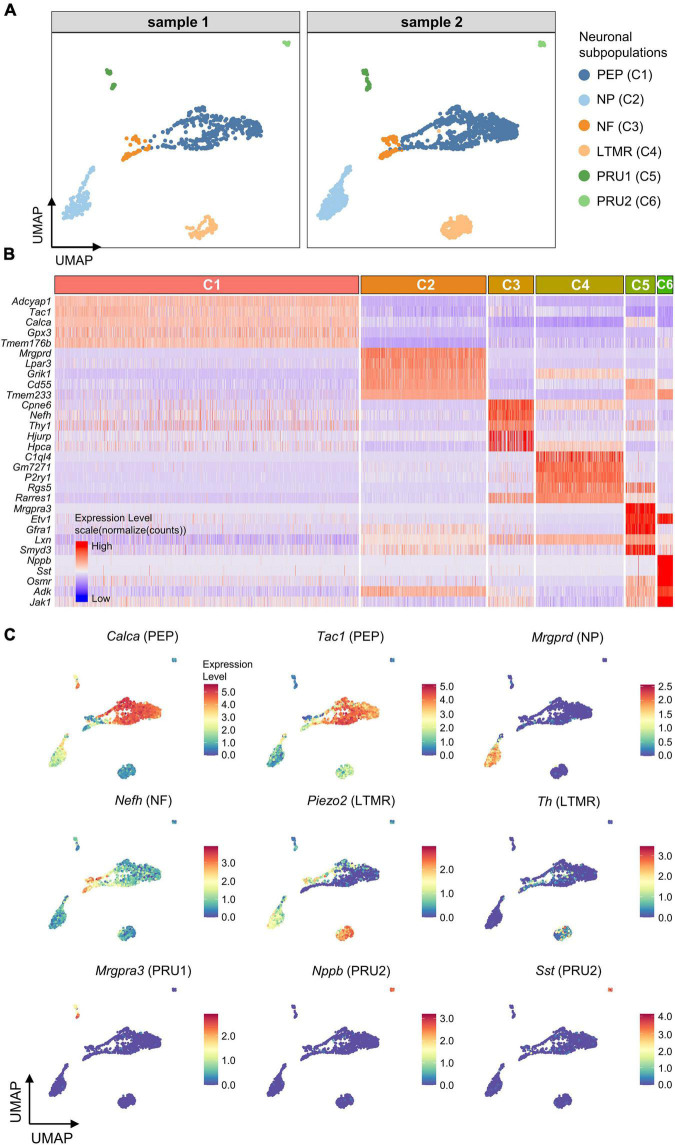
Identification of neuronal subpopulations. **(A)** UMAP projection of neuronal subpopulations. 6 neuronal subpopulations including PEP, NP, NF, LTMR, PRU1, and PRU2 were identified in both samples. Each dot represents one cell; Each color represents one neuronal subpopulation. **(B)** Heatmap showing expression of the top5 marker genes for each neuronal cluster (C1–C6). Colors represents relative expression levels. Red represents high expression and blue low expression. Each color-block above the heatmap represents one neuronal cluster, and its length represents the number of cells per cluster. **(C)** UMAP projection of known marker genes for each neuronal subpopulation in neuronal clusters (C1–C6). Each dot represents one cell. Cells are colored by normalized expression levels (normalized (counts)). Warm colors represent higher gene expression. C1 was identified as PEP by the expression of *Calca* and *Tac1*, C2 was identified as NP by *Mrgprd*, C3 was identified as NF by *Nefh*, C4 was identified as LTMR by *Piezo2* and *Th*, C5 was identified as PRU1 by *Mrgpra3*, and C6 was identified as PRU2 by *Nppb* and *Sst*. PEP, peptidergic neurons; NP, non-peptidergic neurons; NF, large-diameter myelinated neurons; LTMR, low threshold mechanoreceptive unmyelinated neurons; PRU1, pruriceptive neurons type 1; PRU2, pruriceptive neurons type 2.

To further subdivide the neuronal population (C1-6), differentially expressed genes (DEGs) for each neuronal cluster were identified using the Seurat FindAllMarkers function. Six neuronal subpopulations were identified based on the representative DEGs, including peptidergic neurons (PEP; High expression of *Calca*/*Tac1*), non-peptidergic neurons (NP; *Mrgprd*), large-diameter myelinated neurons (NF; *Nefh*), low threshold mechanoreceptive unmyelinated neurons (LTMR, *Piezo2*/*Th*), and pruriceptive (itch-sensing) subpopulations, PRU1 (*Mrgpra3*) and PRU2 (*Nppb*/*Nts*/*Sst*; Figures 3A–C).

**FIGURE 4:**
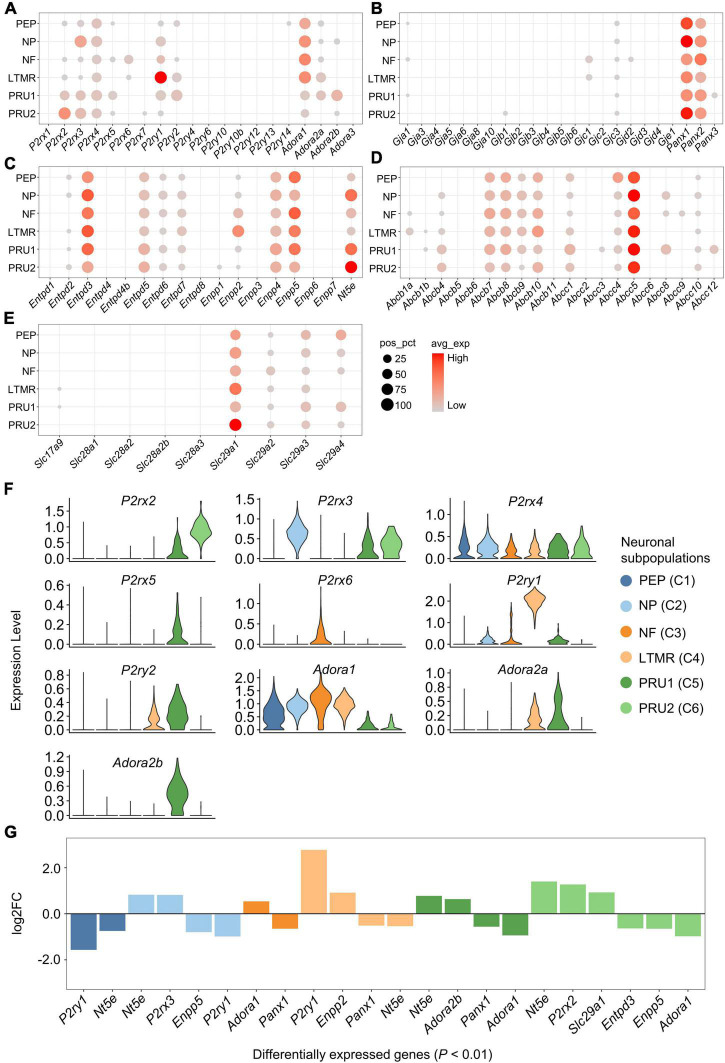
Expression of genes associated with purinergic signaling in neuronal subpopulations. **(A–E)** Bubble plot showing the expression of genes described above in neuronal subpopulations. Bubble size represents percentage of cells with positive expression (pos_pct), and bubble color represents average normalized expression levels (avg_exp). Bubbles with pos_pct < 5% are not shown. **(F)** Violin plots showing the distribution of partial purinergic receptor-encoding genes in distinct neuronal subpopulations. Violin height represents normalized expression level [normalized (counts)], and violin width represents density distribution. Violin plots were color-coded based on neuronal subpopulations. **(G)** Bar plot showing significant differences in gene expression among neuronal subpopulations (Wilcoxon Rank Sum test; *P* < 0.01 was considered statistically significant). The bars are color-coded by neuronal subpopulations, and bar height represents log2 fold change (log2FC). Genes with log2FC > 0.5 and *P* < 0.01 are shown.

We investigated the expression and distribution of genes associated with purinergic signaling in each neuronal subpopulation (Figures 4A–F). Genes encoding purinergic receptors were found differentially expressed in neuronal subpopulations (Figures 4A,F). *P2rx2* was expressed significantly greater in PRU2, *P2rx3* in NP, *P2ry1* in LTMR, *Adora1* in NF, and *Adora2b* in PRU1 (log_2_FC > 0.5 and *P* < 0.01; Figures 4F,G). However, *P2rx4* expression was almost equal in different subpopulations.

In addition, several genes encoding eATP metabolism-associated enzymes or NTs were differentially expressed among the subpopulations. *Nt5e* had significantly higher expression in NP, PRU1 and PRU2, *Enpp2* in LTMR, and *Slc29a1* in PRU2 (log_2_FC > 0.5 and *P* < 0.01; Figures 4C,E,G). Among genes encoding Cxs, Panxs or ABCTs, no significantly differently expressed genes was detected among neuronal subpopulations (log_2_FC < 0.5; Figures 4B,D,G).

### Expression of genes associated with purinergic signaling in glial subpopulations

**FIGURE 5:**
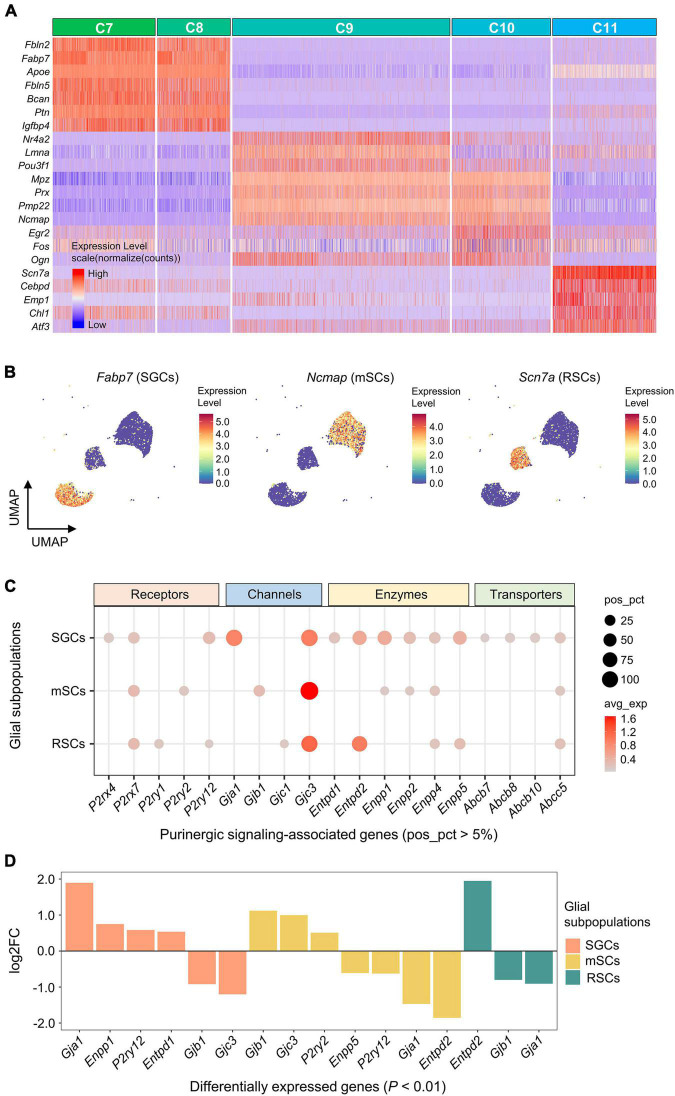
Expression of genes associated with purinergic signaling in glial subpopulations. **(A)** Heatmap showing expression of representative marker genes for each glial cluster (C7–C11). Heatmap colors represent relative expression levels. Red represents high expression and blue low expression. Glial clusters were color-coded above the heatmap. **(B)** UMAP projection of known marker genes for SGCs, mSCs, and RSCs in glial clusters (C7–C11). Each dot represents one cell. Cells are colored by gene expression levels. Levels are normalized expression counts. Warm colors (red and yellow) represent high expression, and cool colors (light and dark blue) represent low expression. C7 and C8 were identified as SGCs by the expression of *Fabp7*, C9 and C10 were identified as mSCs by *Ncmap*, and C11 was identified as RSCs by *Scn7a*. **(C)** Bubble plot showing genes encoding purinergic complex (including receptors, channels, enzymes, and transporters) in glial subpopulations. **(D)** Bar plot showing differential expression of these genes among glial subpopulations (Wilcoxon Rank Sum test; *P* < 0.01 was considered statistically significant). The bars are color-coded by glial subpopulations, and bar height represents log2 fold change (log2FC). Genes with log2FC > 0.5 and *P* < 0.01 are shown. SGCs, satellite glial cells; mSCs, myelinating Schwann cells; RSCs, Remak Schwann cells.

The highest DEGs for each glial cluster (C7-11) were identified and visualized (Figure 5A). Based on the expression of known marker genes, C7 and C8 were identified as satellite glial cells (SGCs; *Fabp7*), C9 and C10 were identified as myelinating Schwann cells (mSCs; *Ncmap*), and C11 was identified as Remak Schwann cells (RSCs; *Scn7a*) (Figure 5B).

Five receptor-encoding genes were positively expressed in glial subpopulations (pos_pct > 5%). Among these, *P2rx7* was positively expressed in all 3 glial subpopulations, *P2ry12* in SGCs and RSCs, *P2rx4* in SGCs, *P2ry2* in mSCs, and *P2ry1* in RSCs (Figure 5C). Differential gene expression analysis showed that *P2ry2* was expressed significantly greater in mSCs, and *P2ry12* in SGCs (log_2_FC > 0.5 and *P* < 0.01; Figure 5D).

Among genes encoding Cxs, *Gjc3* was positively expressed in all 3 glial subpopulations (47.3% of SGCs, 61.4% of mSCs and 43.6% of RSCs), and had significantly higher expression in mSCs (log_2_FC > 0.5 and *P* < 0.01; Figures 5C,D). In addition, *Gja1* was expressed significantly more in 45.3% of SGCs, and *Gjb1* in 11.5% of mSCs (log_2_FC > 0.5 and *P* < 0.01).

Genes encoding E-NTPDase or ENPP were also found differentially expressed among glial subpopulations (Figures 5C,D). Differential gene expression analysis revealed that *Entpd2* was expressed significantly greater in 36.8% of RSCs, and *Enpp1* in 27.3% of SGCs (log_2_FC > 0.5 and *P* < 0.01).

### Expression of genes associated with purinergic signaling in immune cell subpopulations

**FIGURE 6:**
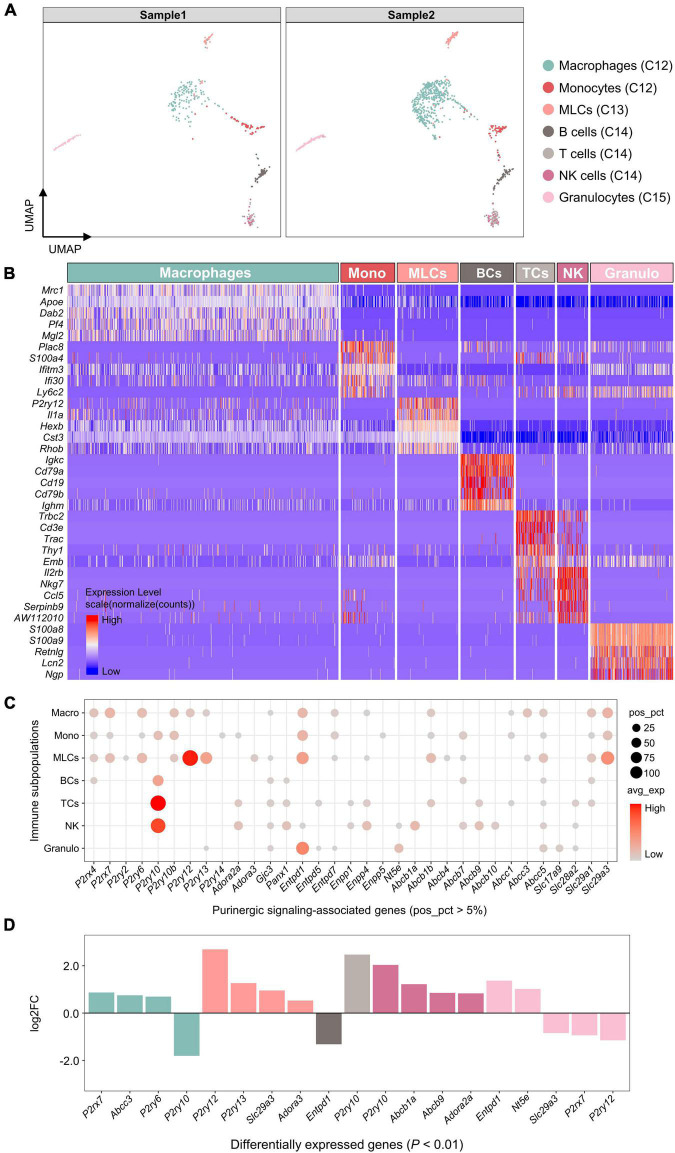
Expression of genes associated with purinergic signaling in immune cell subpopulations. **(A)** UMAP plot showing SingleR automatic annotations. Each dot represents one cell. Cells are colored by SingleR annotations. **(B)** Heatmap showing expression of top 5 marker genes for each immune cell subpopulation annotated by SingleR. Heatmap colors represent relative expression levels. Red represents high expression and blue low expression. Immune cell subpopulations were color-coded above the heatmap. **(C)** Bubble plot showing expression of purinergic signaling-associated genes in immune cell subpopulations. **(D)** Bar plot showing differential expression of these genes among immune cell subpopulations (Wilcoxon Rank Sum test; *P* < 0.01 was considered statistically significant). Genes with log2FC > 0.5 and *P* < 0.01 are shown.

Based on SingleR automatic annotations, 7 immune cell subpopulations were identified, including macrophages, monocytes, microglia-like cells (MLCs), B cells, T cells, NK cells and granulocytes (Figure 6A). The highest DEGs for each immune cell subpopulation were consistent with known canonical markers (Figure 6B).

Among genes encoding purinergic receptors, *P2rx4*, *P2rx7*, *P2ry6*, *P2ry10b*, *P2ry12*, and *P2ry13* were found to be positively expressed in macrophages (pos_pct > 5%; Figure 6C), of which *P2rx7* and *P2ry6* showed significantly higher expression compared with the other immune cells (log_2_FC > 0.5 and *P* < 0.01; Figure 6D). MLCs shared a similar expression pattern with macrophages, and showed significantly higher expression of *P2ry12*, *P2ry13*, and *Adora3* than the other immune cells. Both monocytes and lymphocytes expressed *P2ry10* positively, of which T cells and NK cells showed significantly higher *P2ry10* expression than the other immune cells.

In addition, macrophages significantly expressed *Abcc3*, MLCs significantly expressed *Slc29a3*, NK cells significantly expressed *Abcb1a* and *Abcb9*, and granulocytes significantly expressed *Entpd1* and *Nt5e* (log_2_FC > 0.5 and *P* < 0.01; Figure 6D).

## Discussion

Although many aspects of purinergic signaling have been elucidated, currently little is known about the transcriptional features of purinergic signaling in TG. In recent years, scRNA-seq and single nucleus RNA sequencing technologies have been used to investigate heterogeneity of peripheral ganglia (Usoskin et al., 2015; Nguyen et al., 2017; Mapps et al., 2022; Yang et al., 2022) and transcriptional features of functional gene sets such as transcription factors at the single cell level (Paul et al., 2017; Li et al., 2021). In this study, we revealed the shared and unique expression patterns of genes associated with purinergic signaling in distinct cell types, suggesting that the multidirectional action of purinergic signaling in TG is cell-type specific.

### Purinergic receptors

Purinergic receptor activation up-regulates intracellular Ca^2+^ concentration *via* P2XR-mediated extracellular Ca^2+^ influx and P2YR-/P1R-mediated Ca^2+^ release from the calcium stores (North, 2002; Abbracchio et al., 2006; Antonioli et al., 2013). Purinergic receptors expressed in the TG can be greatly up-regulated following orofacial inflammation and/or nerve injury, contributing to the initiation and maintenance of orofacial allodynia and hyperalgesia (Shinoda et al., 2005, 2007; Katagiri et al., 2012; Ito et al., 2013; Yin et al., 2021). *P2rx3*, the gene that encodes P2 × 3Rs, was mainly expressed in neurons, and was virtually absent in other cell types. Among neuronal subpopulations, *P2rx3* was expressed in 97.6% of NP, 22.7% PEP, and 12.1% NF, which was consistent with previous rat studies (Ambalavanar et al., 2005; Staikopoulos et al., 2007). In addition, we found that *P2rx3* was also widely detected in PRU1 and PRU2, indicating its potential role in pruriception.

Unlike *P2rx3*, *P2rx4* was expressed not only in neurons but also in SGCs and macrophages. In addition, we found that there was no significant difference in *P2rx4* expression among neuronal subpopulations, suggesting that P2 × 4R function is relatively conserved. Several studies have revealed that P2 × 4R is expressed in microglia of spinal trigeminal nucleus (STN), and up-regulated in the chronic migraine models, which may play important roles in central sensitization and pathogenesis of migraine chronicity (Liu et al., 2018; Long et al., 2018). Moreover, P2 × 4R located in DRG or local tissues has been shown to be involved in inflammatory or neuropathic pain (Ulmann et al., 2010; Wang et al., 2020). We speculate that the data about the roles of P2 × 4R obtained in the spinal system could also be operative in the trigeminal system, which would be needed to determine.

*P2rx7* was mainly expressed in glial cells and macrophages, whereas is barely expressed in TG neurons, which is consistent with previous studies (Kushnir et al., 2011; Nowodworska et al., 2017). In addition, we found that *P2rx7* was expressed at similar levels in the 3 glial subpopulations, including SGCs, mSCs, and RSCs. Among immune cell subpopulations, *P2rx7* was expressed not only in macrophages but also in MLCs and monocytes. There is evidence that P2 × 7R in the TG or STN is involved in orofacial pain (Currò et al., 2020; Yin et al., 2021). However, given the cell type-specific expression patterns of P2XRs, there may be differences in mechanisms by which P2XR subtypes promote orofacial pain.

P2YRs are subdivided into G_*q*_-coupled P2Y1-like receptor subtypes (including P2Y1, P2Y2, P2Y4, P2Y6, and P2Y11R) and G_*i*_-coupled P2Y12-like receptor subtypes (including P2Y12, P2Y13, and P2Y14R) (Abbracchio et al., 2006). We found that *P2ry1* was expressed in 40.8% of neurons and 23.4% of fibroblasts, and *P2ry2* was positively expressed in 22.4% of neurons. Previous studies showed that both P2Y1R and P2Y2R in TG were up-regulated after temporomandibular joint (TMJ) inflammation, and antagonism of P2Y2R significantly inhibited the mechanical allodynia, whereas antagonism of P2Y1R was completely ineffective, indicating the potential role of P2Y2R in orofacial pain (Magni et al., 2015). In this study, we observed high *P2ry1* expression in LTMR neuronal subpopulation that also co-express *Piezo2* and *Th* (encoding tyrosine hydroxylase, a key enzyme mediating the conversion of tyrosine to dopamine), suggesting its potential roles in dopamine release, and this prediction has been validated by a recent study (Wei et al., 2022). In addition, *P2ry1* was also expressed in 62.7% of NP and 66.9% of PRU, which can account for its pronociceptive role in regulating of neuropathic pain (Su et al., 2021a,b). *P2ry2* was mainly expressed in LTMR and PRU1 and was rarely expressed in NP, suggesting that the pro-nociceptive role of P2Y2R is not directly related to NP nociceptive neurons. Further studies are necessary to elucidate the molecular mechanism that responsible for pro-nociceptive effect of P2Y1R and P2Y2R.

Gi-coupled P2Y12-like receptor subtypes were mainly expressed in glial cells and immune cells. Previous studies showed that activation of P2Y12R in SGCs of TG was in involved in orofacial neuropathic pain induced by unilateral lingual nerve injury (Katagiri et al., 2012; Sugawara et al., 2017). In addition, microglia P2Y12R in STN was also verified to be involved in several types of orofacial pain, including migraine and tongue cancer pain (Tamagawa et al., 2016; Gölöncsér et al., 2021). In our studies, *P2ry12* was expressed not only in SGCs, but also in macrophages and MLCs, suggesting that the pro-nociceptive effect of P2Y12R may not only rely on the glia-neuron interactions but also on the neuroimmune communication. Moreover, we identified a microglia-like immune cell subpopulation (MLCs) that could be characterized by known marker genes of microglia, including *P2ry12* and *Tmem119* (Li et al., 2019). Although microglia have been considered the CNS-resident phagocytic cells, several studies have provided histochemical evidence for microglia-like macrophages in TG (Glenn et al., 1993; Mori et al., 2003).

Increased eATP following inflammation or tissue injury may be converted to adenosine which exerts complex pro- or anti-nociceptive effects *via* P1Rs activation (Sawynok and Liu, 2003). Growing evidence has shown that A2AR (encoded by *Adora2a*) exhibits the pro-nociceptive effect, whereas A1R (encoded by *Adora1*) acts as an anti-nociceptive target in orofacial pain. Our results showed that *Adora1* was widely expressed in almost all neuronal subpopulations and was more highly expressed in NF neurons, whereas *Adora2a* was mainly expressed in LTMR and PRU1. In addition, *Adora2b* was expressed specifically in PRU1 neurons, whereas *Adora3* was expressed in less than 5% of each neuronal subpopulation. Although *Adora3* was detected at relatively low levels, their roles in mouse TG cannot be discounted. Several studies have shown that A3AR (encoded by *Adora3*) activation in the spinal cord or brain contributes to the alleviation of neuropathic pain induced by paclitaxel, cisplatin or chronic constriction injury of the sciatic nerve (Janes et al., 2014; Ford et al., 2015; Little et al., 2015; Singh et al., 2022). In addition, A3ARs were expressed in rat DRG neurons, and A3AR activation inhibited N-type Ca2^+^ currents and neuronal excitability, which could be independent mechanisms for A3ARs in anti-nociception (Coppi et al., 2019). However, few evidence showed its anti-nociceptive role in orofacial region. Further studies are needed to elucidate the roles of adenosine receptors in orofacial pain.

### Cxs and Panx channels

Increased ATP levels following inflammation and/or tissue injury initiates the purinergic signaling. Both Cxs and Panx channels are transmembrane proteins, mediating the release of ATP *via* their central pores (Taruno, 2018). In addition, the Cxs can form gap junction between neurons and SGCs, mediating the neuron-glia interactions (Spray et al., 2019). The pro-nociceptive properties of Panx1 (encoded by *Panx1*) and Cx43 (encoded by *Gja1*) have been demonstrated (Kaji et al., 2016; Kurisu et al., 2022). Our results showed that the expression of *Panx1* were high in neurons, whereas *Gja1* were low in this population. In contrast, in glial cells, the expression of *Panx1* was nearly absent, whereas *Gja1* was highly expressed in SGCs. In addition, we found that *Panx2* was highly expressed in neurons, and *Gjc3* (encoding Cx29) was highly expressed among SGCs and Schwann cells, which has never been reported before. Further studies are needed to identify their roles in neuron-glia interactions and orofacial pain.

### Extracellular adenosine triphosphate metabolism-associated enzymes

eATP metabolism-associated enzymes can hydrolyze eATP to adenosine, thereby mediating the attenuation of purinergic signaling and the enhancement of adenosine signaling. E-NTPDase family can catalyze the conversion of eATP and/or adenosine diphosphate (ADP) to adenosine monophosphate (AMP) (Yegutkin, 2014). Our results showed that *Entpd3* was specifically expressed in TG neurons, which is consistent with a previous study (Ma et al., 2016). In addition, we found that *Entpd1* was mainly expressed in immune cells and endothelial cells, *Entpd2* was mainly expressed in glial cells and fibroblasts, and *Entpd5*-*7* were mainly expressed in neurons. Among glial subpopulations, SGCs and RSCs expressed *Entpd2*, but not mSCs. Among immune cell subpopulations, macrophages and MLCs expressed *Entpd1*, but not lymphocytes.

ENPP family mediate the conversion of eATP to AMP and PPi, and NT5E mediates the conversion of AMP to adenosine (Yegutkin, 2014). A previous study showed that administration of purified NT5E inhibited the mechanical and thermal hyperalgesia in inflammatory or peripheral neuropathic pain models (Sowa et al., 2010). The expression of NT5E in TG nociceptive neurons has been demonstrated (Liu et al., 2017). Our results showed that *Nt5e* was positively expressed in 56.0% of neurons and 19.9% of fibroblasts, while it was barely expressed in glial cells or immune cells. Among the different subpopulations of neurons, NP and PRU neurons showed high expression of *Nt5e*. Among genes encoding ENPP, *Enpp1* was mainly expressed in SGCs, *Enpp2* was mainly expressed in fibroblasts, and *Enpp4* and *Enpp5* were mainly expressed in neurons. Here, we demonstrate that genes encoding eATP metabolism-associated enzymes are differentially expressed within distinct cell types, which suggests that the capacity to hydrolyze eATP varies among the different cell types.

### Adenosine triphosphate and adenosine transporters

VUNT-dependent vesicular exocytosis represents a critical pathway for eATP release. Previous studies showed that VNUT was predominantly located on the small- and medium-diameter neurons in rat DRG (Nishida et al., 2014) and was up-regulated in neurons of rat TG after tooth extraction (Goto et al., 2017), suggesting its role in orofacial pain. However, *Slc17a9* was not detected in any of the TG cell populations in mice, possibly due to low transcriptional activity of *Slc17a9* under physiological conditions or species difference.

Extracellular adenosine is regulated by NTs, which are membrane transport proteins that mediate the bidirectional diffusion of adenosine (Kong et al., 2004). Previous studies showed that ENT1 was highly expressed in DRG peptidergic neurons, and other neuronal subpopulations including small-diameter nociceptive neurons and large-diameter neurons were also expressed ENT1 positively (Governo et al., 2005). We found that ENT1-encoding gene, *Slc29a1*, was expressed in 76.9% of TG neurons and 15.3% of macrophages, but not in glial cells. Other genes encoding ENTs including *Slc29a2*, *Slc29a3*, and *Slc29a4* were also expressed in neurons. Besides neuron expression, *Slc29a3* was expressed in macrophages, monocytes and MLCs. These results suggest that TG neurons and macrophages are major contributors to adenosine release and reuptake. There is evidence that inhibition of ENT1 exerts an anti-nociceptive effect in inflammatory paw pain models (Maes et al., 2012). Further studies are needed to clarify the roles of ENT family in orofacial nociception and chronic pain.

While this study provides a comprehensive transcriptomic description of the purinergic signaling in TG cells, there are limitations. First, preparation of single-cell suspension from TG inevitably led to neuronal injury, and this might result in transcriptomic changes involving nerve damage and repair. Whether this process involves in the purinergic signaling is unknown. Second, due to the presence of vasculature in TG, whether certain immune cell types, such as macrophages, B cells and T cells are blood-derived or tissue-resident cannot be determined. Nonetheless, we also cannot rule out a role for these cells in TG. Third, this study based on the scRNA-seq to analyze the mRNA expression level of purinergic signaling-associated genes has not been verified at protein levels. Further studies are needed to validate these findings.

## Data availability statement

The datasets presented in this study can be found in online repositories. The names of the repository/repositories and accession number(s) can be found below: www.ncbi.nlm.nih.gov/geo/, GSE213105.

## Ethics statement

The animal study was reviewed and approved by Animal Care Committee for the Care and Use of Laboratory Animals of Sun Yat-sen University (No. SYSU-IACUC-2020-000245).

## Author contributions

SJ: conceptualization, formal analysis, and writing—original draft. JL: data curation, visualization, and software. YC: methodology, visualization, writing—review, and editing. QL: resources, writing—review, and editing. LM: resources and writing—original draft. WF: supervision, writing—review and editing, project administration, and funding acquisition. All authors contributed to the article and approved the submitted version.

## Footnotes

1
https://www.sevenbridges.com

2
https://www.ncbi.nlm.nih.gov/geo/query/acc.cgi?acc=GSE186421

3
http://biocc.hrbmu.edu.cn/CellMarker/

4
https://panglaodb.se/
