# Supplementary material for: Single-cell RNA sequencing reveals distinct transcriptional features of the purinergic signaling in mouse trigeminal ganglion
Source: Front Mol Neurosci. 2022 Oct 13;15:1038539. doi: 10.3389/fnmol.2022.1038539 (PMC9606672; doi:10.3389/fnmol.2022.1038539)
Supplement: Supplementary file 1 [file Table_1.DOCX]

**Supplementary Table S1 Gene symbols of marker genes and corresponding protein names**

| **Gene symbol** | **Protein name** |
| --- | --- |
| *Tubb3* | Tubulin beta-3 chain |
| *Rbfox3* | Rna binding protein, fox-1 homolog (c. elegans) 3 |
| *Plp1* | Myelin proteolipid protein |
| *Ptprc* | Protein tyrosine phosphatase, receptor type, c |
| *Dcn* | Decorin |
| *Col1a1* | Collagen alpha-1(I) chain |
| *Flt1* | Vascular endothelial growth factor receptor 1 |
| *Pecam1* | Platelet endothelial cell adhesion molecule |
| *Adcyap1* | Pituitary adenylate cyclase-activating polypeptide |
| *Tac1* | Protachykinin-1 |
| *Calca* | Calcitonin |
| *Gpx3* | Glutathione peroxidase 3 |
| *Tmem176b* | Transmembrane protein 176B |
| *Mrgprd* | Mas-related G-protein coupled receptor member D |
| *Lpar3* | Lysophosphatidic acid receptor 3 |
| *Grik1* | Glutamate Ionotropic Receptor Kainate Type Subunit 1 |
| *Cd55* | CD55 molecule, decay accelerating factor for complement |
| *Tmem233* | Transmembrane protein 233 |
| *Cpne6* | Copine-6 |
| *Nefh* | Neurofilament, heavy polypeptide |
| *Thy1* | Thymus cell antigen 1, theta |
| *Hjurp* | Holliday junction recognition protein |
| *Hpca* | Neuron-specific calcium-binding protein hippocalcin |
| *C1ql4* | Complement component 1, q subcomponent-like 4 |
| *P2ry1* | Purinergic receptor p2y, g-protein coupled 1 |
| *Rgs5* | Regulator of G-protein signaling 5 |
| *Rarres1* | Retinoic acid receptor responder (tazarotene induced) 1 |
| *Mrgpra3* | Mas-related G-protein coupled receptor member A3 |
| *Etv1* | ETS translocation variant 1 |
| *Gfra1* | Glial cell line derived neurotrophic factor family receptor alpha 1 |
| *Lxn* | Latexin |
| *Rgs5* | Regulator of G-protein signaling 5 |
| *Nppb* | Natriuretic peptides B |
| *Sst* | Somatostatin |
| *Osmr* | Oncostatin-M-specific receptor subunit beta |
| *Adk* | Adenosine kinase |
| *Jak1* | Tyrosine-protein kinase jak1 |
| *Piezo2* | Piezo-type mechanosensitive ion channel component 2 |
| *Th* | Tyrosine 3-monooxygenase |
| *Fbln2* | Fibulin-2 |
| *Fabp7* | Fatty acid-binding protein, brain |
| *Apoe* | Apolipoprotein E |
| *Fbln5* | Fibulin-5 |
| *Bcan* | Brevican core protein |
| *Scd1* | Stearoyl-coa desaturase (delta-9 desaturase) |
| *Ptn* | Pleiotrophin |
| *Ednrb* | Endothelin receptor type B |
| *Ptprz1* | Receptor-type tyrosine-protein phosphatase zeta |
| *Slc4a4* | Solute carrier family 4 (sodium bicarbonate cotransporter), member 4 |
| *Igfbp4* | Insulin-like growth factor-binding protein 4 |
| *Aldoc* | Fructose-bisphosphate aldolase, class i |
| *Mmd2* | Monocyte to macrophage differentiation-associated 2 |
| *Cst3* | Cystatin-C |
| *Acsbg1* | Acyl-coa synthetase bubblegum family member 1 |
| *Nr4a2* | Nuclear receptor subfamily 4 group A member 2 |
| *Lmna* | Prelamin-A/C |
| *Pou3f1* | POU domain, class 3, transcription factor 1 |
| *Mpz* | Myelin protein zero |
| *Prx* | Periaxin |
| *Ncmap* | Noncompact myelin-associated protein |
| *Fxyd6* | FXYD domain-containing ion transport regulator 6 |
| *Cdkn1a* | Cyclin-dependent kinase inhibitor 1 |
| *Pllp* | Plasma membrane proteolipid |
| *Cldn19* | Claudin-19 |
| *Pmp22* | Peripheral myelin protein 22 |
| *Egr2* | E3 SUMO-protein ligase EGR2 |
| *Drp2* | Dystrophin-related protein 2 |
| *Fos* | Proto-oncogene c-Fos |
| *Ogn* | Osteoglycin (osteoinductive factor, mimecan) |
| *Ugt8a* | 2-hydroxyacylsphingosine 1-beta-galactosyltransferase |
| *Egr1* | Early growth response protein 1 |
| *Scn7a* | Sodium channel, voltage-gated, type vii, alpha |
| *Emp1* | Epithelial membrane protein 1 |
| *Chl1* | Neural cell adhesion molecule L1-like protein |
| *Atf3* | Cyclic AMP-dependent transcription factor ATF-3 |
| *Cdh19* | Cadherin 19, type 2 |
| *Ank3* | Ankyrin 3, epithelial |
| *Abca8b* | ATP-binding cassette sub-family A member 8-B |
| *Ifitm3* | Interferon-induced transmembrane protein 3 |
| *Matn2* | Matrilin-2 |
| *Mrc1* | Macrophage mannose receptor 1 |
| *Apoe* | Apolipoprotein E |
| *Dab2* | Disabled 2, mitogen-responsive phosphoprotein |
| *Pf4* | Platelet factor 4 |
| *Mgl2* | Macrophage galactose N-acetyl-galactosamine specific lectin 2 |
| *Plac8* | Placenta-specific gene 8 protein |
| *S100a4* | S100 calcium binding protein A4 |
| *Ifitm3* | Interferon-induced transmembrane protein 3 |
| *Ifi30* | Gamma-interferon-inducible lysosomal thiol reductase |
| *Ly6c2* | Lymphocyte antigen 6 complex, locus C2 |
| *P2ry12* | Purinergic receptor p2y, g-protein coupled 12 |
| *Il1a* | Interleukin-1 alpha |
| *Hexb* | Beta-hexosaminidase subunit beta |
| *Cst3* | Cystatin-C |
| *Rhob* | Rho-related GTP-binding protein RhoB |
| *Cd79a* | B-cell antigen receptor complex-associated protein alpha chain |
| *Cd19* | B-lymphocyte antigen CD19 |
| *Cd79b* | B-cell antigen receptor complex-associated protein beta chain |
| *Cd3e* | T-cell surface glycoprotein CD3 epsilon chain |
| *Trac* | ATP-binding cassette, subfamily g (white), member 5 (sterolin 1) |
| *Thy1* | Thymus cell antigen 1, theta |
| *Emb* | Embigin |
| *Il2rb* | Interleukin-2 receptor subunit beta |
| *Nkg7* | Protein NKG7 |
| *Ccl5* | Chemokine (c-c motif) ligand 5 |
| *Serpinb9* | Serine (or cysteine) peptidase inhibitor, clade B, member 9 |
| *AW112010* | Expressed sequence AW112010 |
| *S100a8* | Protein S100-A8 |
| *S100a9* | Protein S100-A9 |
| *Retnlg* | Resistin-like gamma |
| *Lcn2* | Neutrophil gelatinase-associated lipocalin |
| *Ngp* | Neutrophilic granule protein |

**Supplementary Table S2 Gene symbols associated with purinergic signaling and corresponding protein names**

| **Gene symbol** | **Protein name** |
| --- | --- |
| *P2rx1* | Purinergic receptor p2x, ligand-gated ion channel, 1 |
| *P2rx2* | P2X purinoceptor 2 |
| *P2rx3* | P2X purinoceptor 3 |
| *P2rx4* | Purinergic receptor p2x, ligand-gated ion channel 4 |
| *P2rx5* | P2X purinoceptor |
| *P2rx6* | Purinergic receptor p2x, ligand-gated ion channel, 6 |
| *P2rx7* | Purinergic receptor p2x, ligand-gated ion channel, 7 |
| *P2ry1* | Purinergic receptor p2y, g-protein coupled 1 |
| *P2ry2* | Purinergic receptor p2y, g-protein coupled 2 |
| *P2ry4* | Pyrimidinergic receptor p2y, g-protein coupled, 4 |
| *P2ry6* | Pyrimidinergic receptor p2y, g-protein coupled, 6 |
| *P2ry10* | Purinergic receptor p2y, g-protein coupled 10 |
| *P2ry10b* | Purinergic receptor P2Y, G-protein-coupled 10B |
| *P2ry12* | Purinergic receptor p2y, g-protein coupled 12 |
| *P2ry13* | Purinergic receptor p2y, g-protein coupled 13 |
| *P2ry14* | Purinergic receptor p2y, g-protein coupled, 14 |
| *Adora1* | Adenosine receptor A1 |
| *Adora2a* | Adenosine receptor A2a |
| *Adora2b* | Adenosine receptor A2b |
| *Adora3* | Adenosine receptor A3 |
| *Gja1* | Gap junction alpha-1 protein |
| *Gja3* | Gap junction alpha-3 protein |
| *Gja4* | Gap junction alpha-4 protein |
| *Gja5* | Gap junction alpha-5 protein |
| *Gja6* | Gap junction alpha-6 protein |
| *Gja8* | Gap junction alpha-8 protein |
| *Gja10* | Gap junction alpha-10 protein |
| *Gjb1* | Gap junction beta-1 protein |
| *Gjb2* | Gap junction beta-2 protein |
| *Gjb3* | Gap junction beta-3 protein |
| *Gjb4* | Gap junction beta-4 protein |
| *Gjb5* | Gap junction beta-5 protein |
| *Gjb6* | Gap junction beta-6 protein |
| *Gjc1* | Gap junction gamma-1 protein |
| *Gjc2* | Gap junction gamma-2 protein |
| *Gjc3* | Gap junction gamma-3 protein |
| *Gjd2* | Gap junction delta-2 protein |
| *Gjd3* | Gap junction delta-3 protein |
| *Gjd4* | Gap junction delta-4 protein |
| *Gje1* | Gap junction epsilon-1 protein |
| *Panx1* | Pannexin-1 |
| *Panx2* | Pannexin-2 |
| *Panx3* | Pannexin-3 |
| *Entpd1* | Ectonucleoside triphosphate diphosphohydrolase 1 |
| *Entpd2* | Ectonucleoside triphosphate diphosphohydrolase 2 |
| *Entpd3* | Ectonucleoside triphosphate diphosphohydrolase 3 |
| *Entpd4* | Ectonucleoside triphosphate diphosphohydrolase 4 |
| *Entpd4b* | Ectonucleoside triphosphate diphosphohydrolase 4 |
| *Entpd5* | Ectonucleoside triphosphate diphosphohydrolase 5 |
| *Entpd6* | Ectonucleoside triphosphate diphosphohydrolase 6, isoform cra_a |
| *Entpd7* | Ectonucleoside triphosphate diphosphohydrolase 7 |
| *Entpd8* | Ectonucleoside triphosphate diphosphohydrolase 8 |
| *Enpp1* | Ectonucleotide pyrophosphatase/phosphodiesterase family member 1 |
| *Enpp2* | Ectonucleotide pyrophosphatase/phosphodiesterase family member 2 |
| *Enpp3* | Ectonucleotide pyrophosphatase/phosphodiesterase family member 3 |
| *Enpp4* | Ectonucleotide pyrophosphatase/phosphodiesterase family member 4 |
| *Enpp5* | Ectonucleotide pyrophosphatase/phosphodiesterase family member 5 |
| *Enpp6* | Ectonucleotide pyrophosphatase/phosphodiesterase family member 6 |
| *Enpp7* | Ectonucleotide pyrophosphatase/phosphodiesterase family member 7 |
| *Nt5e* | 5' nucleotidase, ecto |
| *Abcb1a* | ATP-binding cassette, sub-family b (mdr/tap), member 1a |
| *Abcb1b* | ATP-binding cassette, sub-family b (mdr/tap), member 1b |
| *Abcb4* | Phosphatidylcholine translocator ABCB4 |
| *Abcb5* | ATP-binding cassette, sub-family b (mdr/tap), member 5 |
| *Abcb6* | ATP-binding cassette sub-family B member 6, mitochondrial |
| *Abcb7* | ATP-binding cassette sub-family B member 7, mitochondrial |
| *Abcb8* | ATP-binding cassette sub-family b member 8, mitochondrial |
| *Abcb9* | ATP-binding cassette sub-family B member 9 |
| *Abcb10* | ATP-binding cassette sub-family B member 10, mitochondrial |
| *Abcb11* | ATP-binding cassette, sub-family b (mdr/tap), member 11 |
| *Abcc1* | ATP-binding cassette, subfamily c (cftr/mrp), member 1 |
| *Abcc2* | ATP-binding cassette, sub-family c (cftr/mrp), member 2 |
| *Abcc3* | ATP-binding cassette, sub-family c (cftr/mrp), member 3 |
| *Abcc4* | ATP-binding cassette, sub-family C (CFTR/MRP), member 4 |
| *Abcc5* | ATP-binding cassette, sub-family c (cftr/mrp), member 5 |
| *Abcc6* | ATP-binding cassette, sub-family c (cftr/mrp), member 6 |
| *Abcc8* | ATP-binding cassette, sub-family C (CFTR/MRP), member 8 |
| *Abcc9* | ATP-binding cassette, sub-family c (cftr/mrp), member 9 |
| *Abcc10* | ATP-binding cassette, sub-family c (cftr/mrp), member 10 |
| *Abcc12* | ATP-binding cassette, sub-family c (cftr/mrp), member 12 |
| *Slc17a9* | Mfs transporter, acs family, solute carrier family 17, member 9 |
| *Slc28a1* | Sodium/nucleoside cotransporter |
| *Slc28a2* | Solute carrier family 28 (sodium-coupled nucleoside transporter), member 2 |
| *Slc28a2b* | Sodium/nucleoside cotransporter |
| *Slc28a3* | Solute carrier family 28 (sodium-coupled nucleoside transporter), member 3 |
| *Slc29a1* | Equilibrative nucleoside transporter 1 |
| *Slc29a2* | Equilibrative nucleoside transporter 2 |
| *Slc29a3* | Equilibrative nucleoside transporter 3 |
| *Slc29a4* | Equilibrative nucleoside transporter 4 |
